# Experimental 70% hydrofluoric acid burns: histological observations in an established human skin explants *ex vivo* model

**DOI:** 10.3109/15569527.2010.533316

**Published:** 2010-11-15

**Authors:** François Burgher, Laurence Mathieu, Elian Lati, Philippe Gasser, Laurent Peno-Mazzarino, Joël Blomet, Alan H Hall, Howard I Maibach

**Affiliations:** 1 Scientific Action Group, Prevor Laboratory, Valmondois, France; 2 BIO-EC Laboratory, Longjumeau, France; 3 Toxicology Consulting and Medical Translating Services, Inc., Laramie, Wyoming and Colorado School of Public Health, Denver, Colorado, USA; 4 Department of Dermatology, University of California-San Francisco, San Francisco, California, USA

**Keywords:** Human skin explants model, hydrofluoric acid skin penetration, hydrofluoric acid skin penetration kinetics, hydrofluoric acid burns, histology

## Abstract

**Background:**

Hydrofluoric acid (HF) is particularly dangerous due to the potential for systemic effects and induction of severe skin necrosis through two mechanisms: corrosiveness and local tissue toxicity. In addition, because it is only partially dissociated (p*K*_a_ 3.2), it is capable of penetrating deeply into tissues. There is a lack of experimental studies that objectively characterize the behavior of HF diffusion into human skin, specifically the kinetics of tissue penetration resulting in severe cellular lesions.

**Methodology/principal findings:**

We describe the cutaneous effects of HF using an established *ex vivo* human skin model. The diffusion of 70% HF starts within the first minute of contact at the epidermal surface and after 2min reaches the basal layer. In the subsequent minute, the epidermis is destroyed and lesions appear in the papillary dermis after 4min. Soon after, damage appears in the upper reticular dermis. Thus, 70% HF needs only 5min of contact to completely penetrate human skin explants. This experiment is reproducible and corroborates previous studies and clinical effects reported in accidental HF exposures.

**Conclusion/significance:**

This study shows that the management of HF chemical skin exposure is a question of minutes, especially for initial decontamination. These experimental observations could be useful for objectively comparing skin decontamination methods. Further studies should help to confirm these preliminary results.

## Introduction

**Figure 1:**
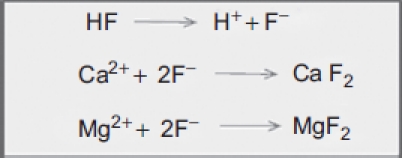
Chemical reactions between fluoride ions and calcium/magnesium. (See colour version of this figure online at www.informahealthcare.com/cot)

Hydrofluoric acid (HF), a particularly dangerous acid because of its corrosiveness and local and systemic toxicity, induces severe tissue necrosis based on the two involved ions: the corrosive hydrogen ion (H^+^) associated with cutaneous [1], ocular [2], and respiratory [3-5] injuries; and the cytotoxic fluoride ion (F^-^) responsible for local and systemic toxicity. In addition, this small molecule, which is only partly dissociated (pK_a_ = 3.2) at the skin surface, is capable of penetrating deeply into the tissues [6]. When dissociation occurs, the skin is partly altered by H^+^, and then the liberated F^-^ ions can develop their toxic properties due to calcium and magnesium chelation [7] (Figure 1). The chelation of calcium and magnesium induces metabolic disorders [8,9] that lead to delayed cellular death and consequent secondary tissue necrosis. System effects [10] are potentially lethal [11-14] depending on the available amount of free fluoride ions.

Since the 1950s [15,16], the management of HF chemical burns has considerably improved. Experimental studies were conducted to further the understanding of the evolution of these burns and to improve their decontamination. Animals studies were performed on pigs [17], rats [18,19], and guinea pigs [20], but no reproducible model was found, especially for high HF concentrations [21].

There is a lack of scientific experimental studies to objectively characterize duration and mode of action of concentrated HF on human skin [22] through understanding of the mechanisms of diffusion and the kinetics of skin penetration or direct observation of the induced cellular lesions.

Presented here are data on the histological lesions induced in human skin *ex vivo* explants exposed to 70% HF. This high concentration was chosen because it is frequently found in laboratories and is widely used in industry. HF is widely used in metallurgy, chemistry, in the paper industry, for engraving (etching) crystal in the glassmaking industry, in analytical chemistry, and as a semiconductor etchant. It is representative of the most dangerous accidental occurrences in cases of splashes due to the severity of burns and potential systemic effects. Histological observations of the stratum corneum, granulosum, and spinosum, the basal and suprabasal layers of the epidermis, and the papillary and reticular dermis were done to demonstrate the severity of the progres-siveness and deterioration evoked by HF burns from the epidermis down to the depth of the dermis.

This study demonstrated the irreversibility of the tissue injury process that became cumulative. This model may contribute to the understanding of the mechanism of HF burns or of those due to other corrosive chemical agents and to the evaluation of decontamination protocols for accidental skin chemical exposures.

## Methods

The tested chemical substance was 70% HF (FLUKA Ref. 47601, Lot 7125 A, titrated to an exact concentration of 73.0%). Twenty-one human skin explants obtained from an abdominoplasty from a 35-year-old woman (Reference P556) were used. Oral informed consent was obtained to use the excised tissue for research purposes. This patient was undergoing an elective cosmetic surgery procedure. The skin tissue excised during this procedure is considered as “medical waste” and may either be disposed of, most often by incineration, or may with the verbal consent of the patient and the operating surgeon, be donated for research purposes. Such tissue is not excised for research purposes. While there is no requirement that the operating surgeon ask the patient, in the interest of Informed Consent, the operating surgeon verbally asked the patient which was her preference. If the patient verbally agreed to the use of the excised tissue, as the tissue would otherwise be destroyed as medical waste, it could without compensation be donated for medical research. In addition, BIO-EC Laboratories had declared these procedures, prior to this study and other studies conducted in a similar manner, to the overall Ethical Committee for the appropriate French geographic sector, Hôpital du Kremelin-Bicêtre, 94270 Le Kremelin Bicêtre, France through the Web site of the French Ministry of Health. As well, BIO-EC had developed prior relationships with clinics and hospitals such that human skin explants, *not* specifically excised for experimentation, could be used in accordance with all applicable ethical principles.

The diameter of each explant was ∼10mm. The explants were preserved in BEM medium (BIO-EC's Explant Medium batch 060208) at 37°C in a moist atmosphere containing 5% CO_2_ for 12-15 h before the study began. The HF acid solution was applied topically on each explant (in triplicate to ensure internal test consistency rather than for any statistical purposes) with a filter paper disk (Medias Filtrans Durieux S.A. reference No. 268, 9 mm diameter) saturated with 30 μL of 70% HF solution. Disks were removed after 20 sec and the reaction was scored histologically at 20 sec, and then at 1, 2, 3, 4, and 5 min after HF-saturated disk removal.

Sampling for explant histology was immediate, just after the end of the exposure.

After 48 h of fixation in Bouin's solution, composed of distilled water (balance), 30% formaldehyde, 5% acetic acid, <4% methyl alcohol, and 1% picric acid (supplied by BIO-EC, batch No. 280299 BHI), the samples were treated by a dehydration and impregnation process performed with a Leica 1020 automatic dehydrator. During this process, water was progressively removed and replaced by ethanol using 70% ethanol, and then a 95% ethanol solution. Ethanol was then replaced by butanol, and the explants finally immersed in a bath containing paraffin at 56°C. This 3-day process included, successively, three baths of 70% ethanol, three baths of 95% ethanol, five baths of butanol, and two baths of paraffin. After preparation, explants were placed in blocks with a Leica EG 1160 coating station; 5-μm slices were made with a Minot-type microtome (Leica 2125) and pressed onto superfrosted silanized glass histology slides. Microscopic observations were performed by optical microscopy with a Leica type DLMB microscope with a 40× objective. Photomicrographs were performed with a CCD Sony DXC 390P camera and stored with Leica IM1000 data archiving software. The observations of general morphology were carried out on paraffin slices dyed with Masson's trichrome, Goldner variant. Cellular structural alterations were searched for in the four main skin layers (i.e. superficial and basal epidermis, papillary and reticular dermis). Because this study resulted in only observational and descriptive data, no statistical analysis was appropriate or could be done.

## Results

All samples of the unexposed control group showed normal morphology. Skin layer thickness of the utilized explants is listed in Table 1. Histological aspects of samples showed that the stratum corneum is more or less thick, moderately laminated, slightly keratinized at the surface and more at its base. The epidermis showed four to five cellular layers.

**Table 1:** Mean thickness of the human skin explants layers.

		Thickness (μm)	
Total skin		3972	
Epidermis	72	Stratum corneum + granulosum + spinosum	57
		Basal layer	15
Dermis	3900	Papillary dermis	248
		Reticular dermis	3652

Note: Standard deviations (SDs) or standard errors of the mean (SEMs) were not calculated.

**Figure 2:**
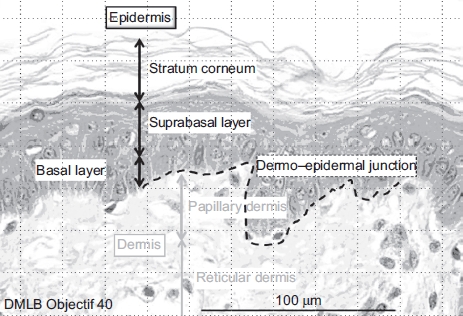
Normal aspect of skin optical microscopy with 40× objective. (See colour version of this figure online at www.informahealthcare.com/cot)

The demarcation of the dermal-epidermal junction was moderate. In the papillary dermis, collagen showed average thickness fibers forming a low-density network. The cellular structures had a normal morphology. In the lower reticular dermis, the cellular structures had normal morphology (Figure 2).

**Figure 3:**
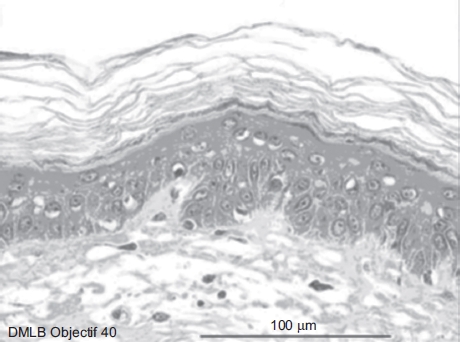
Skin histological aspect after a 20-sec exposure to 30 μL of 70% hydrofluoric acid (HF). No deterioration of the epidermal or dermal structures. (See colour version of this figure online at www.informahealthcare.com/cot)

It was observed that after 20 sec of 70% HF exposure, no deterioration of the epidermal or dermal structures had occurred (Figure 3).

**Figure 4:**
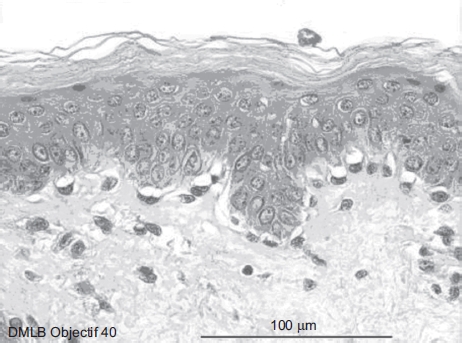
Skin histological aspect at 1 min after a 20-sec exposure to 30 μL of 70% hydrofluoric acid (HF). The epidermis showed four to five cellular layers with a slightly modified morphology. The cells showed gray cytoplasm in the upper layer and the nuclei became pyknotic. The cellular structures of the basal epidermis and throughout the dermis showed normal morphologies. (See colour version of this figure online at www.informahealthcare.com/cot)

At 1min after a 20-sec exposure (Figure 4), the epidermis showed four to five cellular layers with a slightly modified morphology. The cells showed gray cytoplasm in the upper layer and the nuclei became pyknotic. The cellular structures of the basal epidermis and throughout the dermis showed normal morphologies.

**Figure 5:**
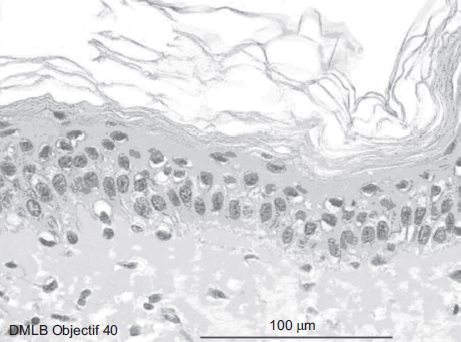
Skin histological aspect at 2 min after a 20-sec exposure to 30 μL of 70% hydrofluoric acid (HF). The skin showed four to five cellular layers with definitely abnormal morphology: cells with nuclei becoming pyknotic, especially in the higher epidermal layers, and the cytoplasm becoming acidophilic as reflected by orange keratinocyte pigmentation. The cellular structures in the dermis showed normal morphology. (See colour version of this figure online at www.informahealthcare.com/cot)

At 2min after a 20-sec exposure, the skin showed four to five cellular layers with definitely abnormal morphology: cells with nuclei becoming pyknotic, especially in the higher epidermal layers, and the cytoplasm becoming acidophilic as reflected by orange keratinocyte pigmentation (Figure 5). The cellular structures in the dermis showed normal morphology.

**Figure 6:**
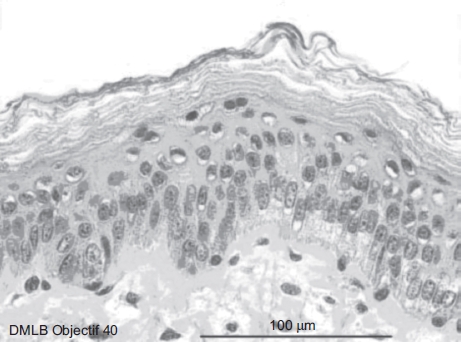
Skin histological aspect at 3 min after a 20-sec exposure to 30 μL of 70% hydrofluoric acid (HF). Lesions of four to five epidermis cellular layers we re characterized by numerous cells with moderately pyknotic nuclei and edema surrounding the nuclei. At the base of the stratum corneum and in the basal epidermis layer, cells showed characteristic cytoplasmic alterations. In the papillary dermis, the cellular structures were slightly pyknotic. The reticular dermis remained normal. (See colour version of this figure online at www.informahealthcare.com/cot)

At 3min after a 20-sec exposure, lesions of four to five epidermis cellular layers were characterized by numerous cells with moderately pyknotic nuclei and edema surrounding the nuclei. At the base of the stratum corneum and in the basal epidermis layer, cells showed characteristic cytoplasmic alterations. In the papillary dermis, the cellular structures were slightly pyknotic. The reticular dermis remained normal (Figure 6).

**Figure 7:**
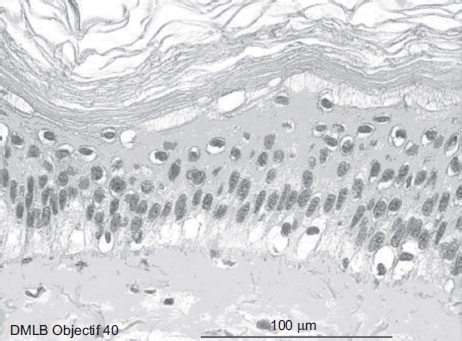
Skin histological aspect at 4 min after a 20-sec exposure to 30 μL of 70% hydrofluoric acid (HF). The same deteriorations in the epidermis were observed compared with those described at 3 min. In the papillary dermis, cells more clearly showed pyknotic nuclei, but the reticular dermis remained normal. (See colour version of this figure online at www.informahealthcare.com/cot)

At 4min after a 20-sec exposure, the same deteriorations in the epidermis were observed compared with those described at 3 min. In the papillary dermis, cells more clearly showed pyknotic nuclei, but the reticu-lar dermis remained normal (Figure 7).

**Figure 8:**
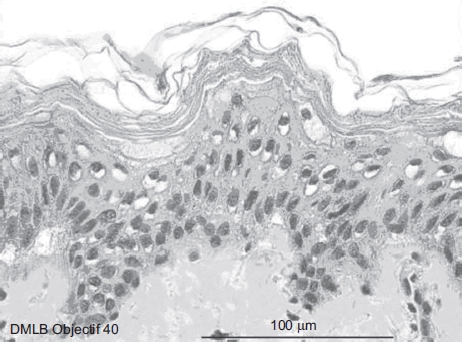
Skin histological aspect at 5 min after a 20-sec exposure to 30 μL of 70% hydrofluoric acid (HF). The same lesions as those detected after 4 min were observed in the epidermis and papillary dermis. However, as the HF had penetrated into the reticular dermis, slightly pyknotic nuclei were observed in cells in this deepest layer. (See colour version of this figure online at www.informahealthcare.com/cot)

Finally, at 5 min after a 20-sec exposure, the same lesions as those detected after 4min were observed in the epidermis and papillary dermis. However, as the HF had penetrated into the reticular dermis, slightly pyknotic nuclei were observed in cells in this deepest layer (Figure 8).

## Discussion

Skin thickness varies considerably between races and age groups, between men and women, and according to different body regions [23-30]. For the epidermis, the chief difference is in the horny layer. The basal cell layer remains constant in nearly all cases [25-27].

The dermis comprises most of the skin thickness and varies more from one skin region to another. Relatively thin in youth, the dermis reaches its maximum thickness between 40 and 50 years of age. Then it decreases until the thickness in old age resembles that in childhood [28].

Table 2 shows the differential published values between maximum and minimum thickness of the skin. In addition, the last line shows the comparative data of the *ex vivo* explants used in the current study. Table 3 shows the differences between Asian and European skin layer thickness. The epidermis thickness also partly depends on the techniques of measurement and/or tissue fixation.

**Table 2:** Comparison between known values of skin thickness.

Authors	Epidermis (μm)	Dermis (μm)	Total thickness (μm)
Cowdry [26]	20–100		
Maximow and Bloom [27]	70–140	1000–3000	Calculated*: 1070–3140
Southwood [28]	20–140	400–2500	Calculated*: 420–3900
Artz et al. [30]	39–64	956–1911	Calculated*: 995–1975
Lee and Hwang [29]	31–637	469–1942	Measured*: 521–1277
Our explants	72	3900	Measured*: 3972

* Measured histologically. There are differences between the calculated values (sum of the minimal and the maximal data) and the measured one because the thinnest epidermis was not necessarily associated with the thinnest dermis and vice versa. Values given are means. Neither SDs nor SEMs were calculated. There data are only intended to show the relative similarity of the human skin explants utilized in this study to published values for normal skin thickness.

**Table 3:** Comparison of thickness of abdomen skin between races.

Abdomen thickness (μm)*	Epidermis	Dermis	% E (E+D)
European	41–40	1640–1492	2.4–2.6
Asian	69	1248	6.0
Our experiment	72	3900 (papillary dermis: 248, reticular dermis: 3652)	1.8

* European values taken from Southwood [28] and Artz et al. [30] versus Asian from Lee and Hwang [29]. Last line: our experimental data.

The kinetics of HF skin penetration is clinically known through observed signs and symptoms during accidental exposures. Based on the analysis of accidents in workers, high concentration HF burns are generally thought to completely develop in the following few minutes after exposure [31]. To improve and further specify immediate first-aid care, it is useful to have a more precise understanding of the development of the initial lesions following HF contact.

The key factors in the development and severity of HF cutaneous burns are concentration, contact time, total body percentage surface area (TBSA) exposed, and skin penetration. However, there is a lack of knowledge on tissue damage regarding the time needed for full-thickness skin penetration of concentrated HF.

The established *ex vivo* human skin explant model utilized in this study allowed real-time histological observation of the diffusion of 70% HF through the skin as manifested by the evolution of cellular and tissue injury. The histological observations offered the possibility of closely following the reaction of the tissue, layer by layer, in the epidermis and dermis after contact with concentrated HF. Human skin explants from all the control samples maintained alive during the experiments (performed in triplicate to ensure internal test consistency) showed normal cellular morphology. For the HF-exposed explants, the lesions were identical on the three series treated in parallel at each stage. Thus, the human skin explant model exposed to 70% HF is reproducible, demonstrating the irreversibility, severity, and rapidity of penetration of 70% HF, and its capacity to cause dermal burns.

In these experimental conditions, 70% HF did not cause immediate massive injury. The cellular alterations first appeared between 20 sec and 1min after a 20-sec exposure. HF penetration through all layers of the human skin explants was observed at 5 min after a 20-sec contact. The onset of epidermal lesions after 1 min of exposure is in accordance with both previously reported experimental data [31] and previouslyreported accidental exposures [32]. The slight cellular deteriorations that appeared in the superficial epidermal layer progress, at 3 min after a 20-sec exposure, to emerging cellular edema in the epidermis and slightly pyknotic cells in the papillary dermis. At 5 min after a 20-sec HF contact, the lesions are clearly obvious in the epidermis and papillary dermis. Lower reticular dermis alterations were still minimal. Tables 4 and 5 summarize these observations.

**Table 4:** Dynamics of appearance of lesions after skin exposure to 70% hydrofluoric acid (HF).

Duration of exposure	Microscopic morphology
1 min	Beginning of the attack in the higher part of the epidermis
2 min	Attack of the basal layer of the epidermis
3 min	Epidermis completely damagedFirst lesions of the papillary dermis (superficial part of the dermis)
4 min	Epidermis completely damagedClear attack of the papillary dermis
5 min	Epidermis completely damagedBeginning of attack of the reticular dermis (deeper layer of the dermis)

**Table 5:** Details of histological cell alterations during a 5-min skin exposure to 70% hydrofluoric acid (HF).

20 sec	Epidermis	GM*
	Papillary dermis	(Good morphology)
	Reticular dermis	
1 min	Epidermis	Slightly PN + AC (Pyknotic nucleus and acidophilic cytoplasm)
	Papillary dermis	GM
	Reticular dermis	
2 min	Epidermis	PN + AC
	Papillary dermis	GM
	Reticular dermis	
3 min	Epidermis	PN + AC
	Papillary dermis	Slightly PN + AC
	Reticular dermis	GM
4 min	Epidermis	PN + AC
	Papillary dermis	
	Reticular dermis	GM
5 min	Epidermis	PN + AC
	Papillary dermis	
	Reticular dermis	Slightly PN + AC

* Cell alterations: good morphology (GM), pyknotic nucleus (PN), and acidophilic cytoplasm (AC).

**Figure 9:**
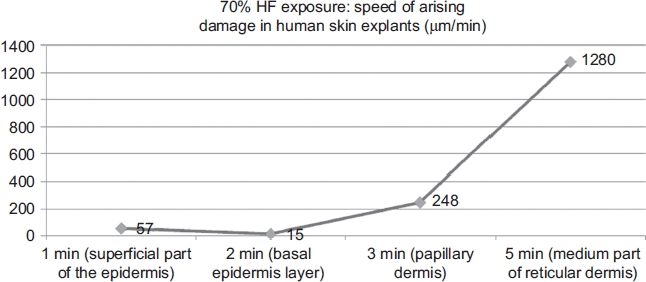
Kinetics of 70% hydrofluoric acid (HF) through human *ex vivo* skin explants. (See colour version of this figure online at www.informahealthcare.com/cot)

From the study presented here, it was possible to estimate the rapidity of emergence of cellular lesions (Figure 9) into each skin layer, as all skin layer thicknesses were measured histologically.

Penetration rates were approximated by histological observations of evolving cellular and tissue damage. The observed penetration rate through the stratum corneum, granulosum, and spinosum layers was -57 μm/min. These data are interesting because the rate-limiting barrier to the absorption of most chemicals is the stratum corneum [33]. Then, the penetration rate slowed to 15 μm/min, and it took about one more minute to cross into the lower basal layer. This decrease in penetration rate maybe due to the specific structure of the acellular dense connective tissue of the basement membrane separating the epidermis from the upper dermis. Afterward, the penetration rate accelerated up to 248 μm/min through the papillary dermis. At the end of the experiment, HF injury was as deep as 1600 μm from the skin surface. In the reticular dermis, the penetration rate reached 1280 μm/min, probably due to the specific composition and density of this layer. Although the cellular alterations were evident (Table 5), tissue structures maintained a coherent appearance, even at 5min after a 20-sec exposure to 70% HF.

The epidermis remained visible by microscopic observation in a human case report with a lethal 60% HF burn, as described by Ohtani et al. [34]. Also, we did not observe massive necrosis (denaturation of the cells and disintegration of the structures) in this human skin *ex vivo* study, as has been frequently described in the clinical setting following accidental cutaneous acid chemical burns in humans [35]. This might be a unique pathological feature of the skin lesions in HF burns. These findings could be due to a specific effect of HF as opposed to other inorganic acids. Compared with a strong acid, such as hydrochloric acid (HCl), HF is 1000 times less dissociated. This is derived from the following formula [36]:

For an acid such as HF:


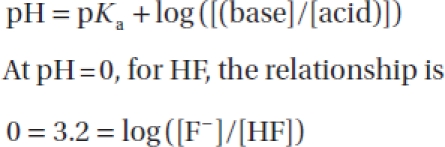


and thus is 10^−3.2^ or ∼10^−3^ = [F^-^]/[HF]

Whereas a strong acid such as HCl is completely dissociated at pH = 0, and therefore:


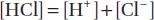


Thus, there is a factor of-1000 between the concentration of F” and the concentration of nondissociated HF.

The observed cellular alterations are correlated with the chemical and physical properties of HF, a particularly dangerous acid, corrosive, and protoplasmic poison, since it is a small molecule (MW= 20) and a partially dissociated acid (pK_a_ = 3.2) at the skin surface.

Although HF is partially dissociated at the skin surface, another portion is nondissociated and can penetrate, and then be dissociated more deeply in the tissue. Previous studies [37,38] suggest that HF is nondissociated at the surface of the skin and can easily penetrate through the epidermis and would easily cross lipid membranes. Matsuno [39] also suggests that nondissociated HF rapidly penetrates the skin. Gutknecht and Walter [40] studied HF transport through lipid bilayer membranes, hypothesizing that F^-^ transport through biological membranes occurs mainly by nonionic diffusion of HF. Membrane permeability of HF ranges from 10^−4^ to 10^−3^ cm/sec, five to seven orders of magnitude higher than the permeability of F^-^ and H^+^.

Dissociation with liberation of F^-^ ions would occur secondarily in deeper tissues. The liberated fluoride ion attacks enzymes and cell membranes [39]. The formation of salts with tissue cations such as calcium and magnesium drives progressive dissociation of HF molecules. The residues are relatively insoluble and stable (p*Ks* CaF_2_= 10.5 and p*Ks* MgF_2_ = 8.2), precipitating within the tissues [41]. Other fluoride salts are much more soluble and dissociable [41], liberating fluoride ion that remains available to react chemically with tissues [42].

In these experimental conditions, HF corrosiveness generates a caustic burn at the epidermal surface, followed by a completely disrupted physiological equilibrium. The observed kinetics of cellular skin damage due to 70% HF demonstrated that initial decontamination is a question of about 1 min to prevent or minimize the severity of HF burns. This experimental model seems to be a useful instrument for further experiments to more completely understand the kinetics and mechanism of cutaneous damage due to chemical agents and for comparing the efficacy of decontamination solutions [43].
