# Corrigendum

**DOI:** 10.3109/15569527.2011.613606

**Published:** 2011-09-23

The publisher would like to apologise on behalf of the authors. Since publication of: Experimental 70% hydrofluoric acid burns: histological observations in an established human skin explants ex vivo model by François Burgher, Laurence Mathieu, Elian Lati, Philippe Gasser, Laurent Peno-Mazzarino, Joël Blomet, Alan H. Hall, and Howard I. Maibach in Cutaneous and Ocular Toxicology, 2011;30(2):100107, the authors have informed us of the following errors:

In the Methods section,

70% HF was applied to the explants on filter paper discs for the following time periods: 20 seconds, 1 minute, 2 minutes, 3 minutes, 4 minutes, and 5 minutes. “Sampling for explant histology was immediate, just at the end of exposure.” That is, at the end of the above time period exposures.

In the Results section,

under “Application of products and washing solutions”, second paragraph, the first sentence should read: “For group F, HF was left in contact with the skin for various times (Table 2) and then the filter paper discs were removed.” Table 2 seems to be correct as published.

under “Histology sampling”, the last sentence of the second paragraph should read: “Then, two explants were sampled and fixed in the same manner with removal of the saturated patches at each of the sampling times: 5, 10, 15, 30 min, 1, 2, and 4h, and 24 h following HF exposure.”

In the Results section,

The 4th paragraph should read: “At 1 min after exposure (Figure 4)...”

The Legend for Figure 4 should read: “Figure 4: Skin histological aspect at 1 minute after exposure...”

The 5th paragraph should read: “At 2 min after exposure...(Figure 5)...”

The Legend for Figure 5 should read: “Figure 5: Skin histological aspect at 2 min after exposure...”

The 6th paragraph should read: “At 3 min after exposure...(Figure 6)...”

The Legend for Figure 6 should read: “Figure 6: Skin histological aspect at 3 min after exposure...”

The 7th paragraph should read: “At 4 min after exposure...(Figure 7)...”

The Legend for Figure 7 should read: “Figure 7: Skin histological aspect at 4 minutes after exposure...”

The 8th paragraph should read: “Finally, at 5 min after exposure...(Figure 8)...”

The Legend for Figure 8 should read: “Figure 8: Skin histological aspect at 5 min after exposure...”

In the Discussion section,

In the 7th paragraph, everywhere the words “after a 20-sec exposure” are incorrect and the correct text should read: “The cellular alterations first appeared between 20 sec and 1 minute after exposure. HF penetration through all layers of the skin was observed at 5 minutes after exposure. ... At 5 min of contact, the lesions are clearly obvious...”

In the 9th paragraph, last sentence should read: “Although the cellular alterations were evident (Table 5), tissue structure maintained a coherent appearance even at 5 minutes after exposure to 70% HF.”

